# The Association Between HIV Testing Modality and Antiretroviral Therapy Initiation Among Men Who Have Sex with Men in Selected Provinces of South Africa

**DOI:** 10.3390/ijerph23020185

**Published:** 2026-01-31

**Authors:** Betty Sebati, Anthony Brown

**Affiliations:** School of Interdisciplinary Research and Graduate Studies, College of Graduate Studies, University of South Africa, Pretoria 0002, South Africa; browna@unisa.ac.za

**Keywords:** HIV testing, HIV testing modalities, antiretroviral therapy initiation, men who have sex with men, South Africa

## Abstract

**Highlights:**

**Public health relevance—How does this work relate to a public health issue?**

- HIV/AIDS remains a major public health issue in South Africa, with key populations such as men who have sex with men experiencing challenges including delayed HIV diagnosis due to structural barriers and stigma.
- This study is focused on important points of the HIV cascade, including HIV testing, which is the first step that leads to the other steps in the cascade and is eventually responsible for us knowing the health outcomes and transmission rates.

**Public health significance—Why is this work of significance to public health?**

- The study identifies HIV testing modalities associated with successful antiretroviral therapy initiation in a key population group.
- The findings of this study may guide efforts to increase early antiretroviral therapy uptake and contribute to reaching the 95-95-95 UNAIDS goals.

**Public health implications—What are the key implications or messages for practitioners, policy makers and/or researchers in public health?**

- There may be a need for a scale-up of differentiated HIV testing modalities (community based, peer-led, etc.), and these may be incorporated into national HIV testing strategies upon the identification of HIV testing modalities associated with ART initiation across various populations.

**Abstract:**

Despite South Africa being the epicentre of HIV, some progress was made in the fight against HIV, i.e., the implementation of HIV programmes, provision of antiretroviral therapy (ART), etc. However, little is known about the association between HIV testing modalities and ART initiation. This study aimed to determine the association between HIV testing modalities and ART initiation among men who have sex with men (MSM) in selected provinces of South Africa. Following a retrospective cohort design, this study analysed programme data on 3345 MSM aged 16 years and older who were living with HIV and eligible for ART initiation. Logistic regression assessed the association between HIV testing modalities and ART initiation, controlling for age group, location, and the COVID-19 period. All analyses were done using SPSS version 30. Significance was set at *p* < 0.05. Participants who tested for HIV using the social network strategy (SNS) (98.6%) or index testing (96.3%) showed the highest proportions of ART initiation. Logistic regression showed that MSM who tested for HIV using the SNS had over 12 times higher odds of initiating ART (aOR = 12.166; 95% CI: 7.617–19.430; *p* < 0.001), compared to those who used a rapid test. A significant association was observed between HIV testing modalities and ART initiation, with SNS and index testing demonstrating higher odds of ART initiation.

## 1. Introduction

Approximately 40.8 million people are estimated to be living with HIV globally [1]. With about eight million people living with HIV (PLHIV), equivalent to approximately 12.7% of the population, and 16.7% being adults aged 15–49 years, South Africa has the highest burden of HIV/AIDS. It further accounts for nearly 20% of HIV cases worldwide [2]. Furthermore, key and vulnerable populations, including female sex workers and men who have sex with men (MSM), are disproportionately affected by HIV, with inadequate access to HIV services due to systematic barriers, stigma and discrimination, among other challenges [3].

There has been progress in the fight against the HIV pandemic. The interventions include the implementation of HIV prevention programmes, expansion of HIV testing services, and provision of antiretroviral therapy (ART), for which South Africa has the largest programme [4,5]. These interventions have greatly improved the management of HIV, although it is still untreatable and life-threatening over time if not well managed [5]. Regardless of the improved efforts put in place for people to test for HIV, one out of every six PLHIV is still not aware of their HIV status worldwide [6,7]. In South Africa, approximately 5–10% of the population are unaware of their HIV status, while 89.6–95% are aware in the 15 years and older age group [2]. Furthermore, nearly 14.7%, 14.4%, 12.7%, 11.9%, 11.4%, 8.5%, and 6% of PLHIV remain unaware of their HIV status in the Gauteng, Western Cape, Mpumalanga, Free State, Limpopo, Northern Cape, and KwaZulu-Natal Provinces of South Africa [2,8].

For HIV status awareness, the first vital step is HIV testing and counselling (HTC) [9]. There are three HIV testing models available based on the service delivery approaches, as well as the settings in which they are provided, i.e., health facility-based, non-health facility-based, and community-based [10]. Health facility-based HIV testing refers to testing conducted in clinical settings including primary health care facilities and hospitals, while non-health facility-based refers to testing done outside of the traditional clinical settings; however, these may not be community-led (i.e., workplace or mobile clinic testing) [10]. Although community-based HIV testing is a category of non-health facility-based testing, it is a specific approach differentiated by its focus on peer leadership, community ownership and involvement, and targeted outreach to key and vulnerable populations such as MSM [10]. Subsequent to HIV testing are ART initiation and linkage to care. Low linkage to care rates upon HIV diagnosis among PLHIV in sub-Saharan Africa have been reported [11]. In South Africa, ART coverage was reported at approximately 79%, with linkage to care estimated at 80% of those diagnosed [1]. In an effort to improve accessibility to HIV testing for those not easily reached or able to access HIV testing services in primary health facilities, various types of non-health facility- and community-based HIV testing strategies have been presented. These can be implemented as promotional campaigns that mostly target populations at greatest risk of HIV, i.e., MSM, and as HIV testing programmes in the workplace or at home, including HIV self-testing [11,12].

A comprehensive meta-analysis including 188 studies reported that community-based HIV testing modalities had higher odds of reaching men as compared to HIV testing based in facilities. Further, linkage to care was also high in this group [13]. Moreover, studies from three sub-Saharan African countries, i.e., South Africa, Tanzania, and Mozambique, reported community-based HIV testing to be linked with decreased linkage to care rates, indicating the necessity of interventions to facilitate this important step [14,15]. There is a possibility of differences in the completion of the HIV care cascade (i.e., ART initiation, retention in care and being virally suppressed) based on the mode of HIV testing. However, there is limited knowledge on the latter [12].

The delayed diagnosis of HIV is seen in the late care engagement and delay in ART initiation [16], which contributes to an unfavourable prognosis for the individual. Thus, implementing new approaches to enhance access to HIV testing, specifically for populations who are not effectively reached by conventional HIV testing modalities, may be necessary. Decentralisation and engaging the community are key to attaining high coverage of HIV care [17]. Although community-based testing has been found to improve uptake of HIV testing [18], it is not clear whether the modality affects the commitment to the cascade of care, with ART initiation being one of the first crucial steps. Hence, this study aims to determine the association between HIV testing modalities and ART initiation among MSM in selected provinces of South Africa. Overall, this study compares four HIV testing modalities (i.e., rapid test, HIV self-screening, index testing, and the social network strategy (SNS)) classified under the community-based HIV testing model, administered through the MSM programme.

## 2. Materials and Methods

### 2.1. Study Design

This study followed a retrospective cohort design, utilising routine programme data from a non-governmental organisation offering HIV prevention services to MSM in selected provinces of South Africa.

### 2.2. Sample Size

This study included programme data involving 3345 MSM aged 16 years and older who were living with HIV and utilising HIV testing and ART initiation services in the selected districts/provinces of South Africa.

### 2.3. Study Population and Setting

The programme was conducted among MSM (i.e., bisexual men, gay men, and other MSM), aged 16 years and above, across three selected provinces and five districts in South Africa. These included two districts in KwaZulu-Natal (i.e., UMgungundlovu, eThekwini), two districts in Gauteng (i.e., Ekurhuleni, Tshwane), and one district in Mpumalanga (i.e., Ehlanzeni).

KwaZulu-Natal, which is the second most populated province (12.3 million people) in South Africa, has been labelled the HIV epicentre of South Africa, with eThekwini and uMgungundlovu recording HIV prevalences of 17.6% and 9.2% respectively [19,20]. Gauteng, which is the smallest province (18,178 km^2^), is the most densely populated (15.9 million) and is the economic engine of the country, with regions such as Tshwane and Ekurhuleni making large contributions to the economy. Gauteng reported an overall HIV prevalence of 11.9%, predominantly affecting adults aged 25–49 years [19,21]. Mpumalanga, the second smallest province (76,495 km^2^), has a provincial population of 5.1 million and 17.4% HIV prevalence, with Ehlanzeni district accountable for 16% [19,22]. The current study focused specifically on MSM who were found to be living with HIV through the programme’s various HIV testing modalities and were eligible for ART initiation in the abovementioned districts within the three provinces.

### 2.4. Data Source

The MSM programme data analysed in the current study was collected from 1 January 2018 to 31 December 2022 in the selected provinces and obtained through approval from a non-governmental organisation in South Africa, which is/was funded by the Centres for Disease Control and Prevention. It offers HIV, tuberculosis, and sexually transmitted infection prevention and treatment services to populations at high risk, including MSM, in partnership with the South African Department of Health primary health care facilities. The programme has thus far been executed in five districts across three provinces of South Africa. The selection was established based on these areas showcasing the highest incidence and prevalence of HIV in the population of interest, i.e., MSM. The primary focus is the prevention of HIV transmission and limiting new HIV cases, as well as a contribution towards ending HIV as a pandemic.

As part of its services, the programme offers HIV testing through various modalities including rapid testing, HIV self-screening, the social network strategy, and index testing as explored in the current study. All four HIV testing modalities were implemented concurrently within the same population (MSM) and time period across all the five districts within the three provinces of the study. Uptake depended on individual preference.

### 2.5. Study Variables

The data collected by the non-governmental organisation was de-identified routine programme data at the individual level, which is reported using the Department of Health’s data collection tools. More information on the variables/indicators collected by the programme can be obtained from Sebati et al. [23]. The data was captured using RedCap v15.0.14 and Three Interlinked Electronic Register (Tier.net) v1.13.3.0 software, the data-capturing platform of the South African Department of Health. Moreover, the collected data is stored in the organisation’s data warehouse for the purposes of cleaning, analysis, and generating reports.

For the current study, the following HIV indicators were considered: HIV testing modalities (i.e., rapid test, HIV self-screening, social network strategy, index testing), HIV test results, and ART initiation. Age group, district, and the COVID-19 period were also included in the analysis.

The main outcome variable was ART initiation, which was defined as having initiated ART within 30 days of HIV diagnosis. The exposure variables, i.e., HIV testing modalities, were defined as follows:

Rapid test: A rapid diagnostic HIV test is used to get preliminary HIV results from as early as 10 min, utilising either oral fluid or a finger prick blood sample.

Social Network Strategy: This strategy leverages existing social networks or relationships to promote HIV testing, wherein trained individuals promote HIV testing to their peers who may be faced with stigma or other barriers limiting them from accessing traditional HIV testing services/health facilities.

Index testing: This is a form of a case-finding HIV testing approach wherein individuals who have been found to be living with HIV are encouraged to refer their sexual partners or needle sharing partners, as well as their families/children whom they may have had blood contact with, for HIV testing.

HIV self-screening: An individual voluntarily collects their oral fluid or blood sample, conducts an HIV test, and interprets it by themselves.

### 2.6. Statistical Analysis

Descriptive statistics were employed to describe the characteristics of the study population. Age was grouped into 16–24 years, 25–34 years, 35–44 years, and 45 years and above. Frequencies and percentages were used to describe the age groups, location, HIV testing modalities, HIV test results, and ART initiation. Bivariate analysis, i.e., a Chi-square test, was used to determine the association between HIV testing modalities (exposure variable) and ART initiation (outcome variable). Logistic regression was applied to determine the impact of HIV testing modalities on ART initiation, controlling for age group, location, and the COVID-19 period in South Africa (March 2020–April 2022, when the national state of disaster was lifted) [24]. This is presented as 95 confidence intervals (CIs) and adjusted odds ratios (aORs) to improve the interpretability All analyses were done using SPSS version 30 (IBM Corp., Armonk, NY, USA). Significance was set at *p* < 0.05.

### 2.7. Ethics Approval

Ethics approval for this study was obtained from the University of South Africa (UNISA) College of Graduate Studies Research Ethics Review Committee (RERC) (Ref number: 9698). Moreover, approval to utilise the programme data was obtained from the non-governmental organisation (Ref number: DSGC-00038).

## 3. Results

Table 1 below shows the characteristics of the MSM included in this study. Most of the MSM were in the 25–34 age group (N = 1678; 50.2%), while the fewest MSM were in the 45+ years age group (N = 327; 9.8%). Most of the MSM were located in the KwaZulu-Natal eThekwini Metropolitan Municipality (N = 985; 29.4%). These were followed by Gauteng’s City of Tshwane and Ekurhuleni Metropolitan Municipalities with 765 (22.9%) and 849 (25.4%) MSM, respectively. The most commonly used method of HIV testing was rapid testing (N = 1721; 51.4%), followed by the SNS (N = 1464; 43.8%). Index testing and HIV self-screening were the least commonly used methods to test for HIV, both contributing 2.4% (N = 80 for both). Most of the MSM living with HIV were initiated on ART within 30 days of HIV diagnosis (N = 2972; 88.8%).

Table 2 presents the association between HIV testing modalities and antiretroviral therapy initiation among men who have sex with men, obtained using the Chi-square test. The Chi-square test showed a statistically significant association between HIV testing modalities and ART initiation (*p* < 0.001). Participants who tested for HIV through the SNS (98.6%) or index testing (96.3%) demonstrated the highest proportions of ART initiation, in comparison to rapid testing (80.3%) and HIV self-screening (86.3%). On the contrary, the proportions of participants who were not initiated on ART were higher among participants who tested for HIV using rapid testing (19.7%) and HIV self-screening (13.8%) than among those who used other HIV testing modalities.

Table 3 presents a logistic regression showing the association between HIV testing modalities and ART initiation among men who have sex with men, adjusted for age group, location, and the COVID-19 period. Using rapid HIV testing as the reference category, the analysis showed a significant association between the HIV testing modality and ART initiation (*p* < 0.001). Further, MSM who tested for HIV using the SNS had over 12 times higher odds of initiating ART (aOR = 12.166; 95% CI: 7.617–19.430; *p* < 0.001), compared to those who had a rapid test. Participants who had an index test had 3.719 higher odds of initiating ART (aOR = 3.719; 95% CI: 1.142–12.109; *p* = 0.029). There was a positive but non-significant association between HIV self-screening and ART initiation (*p* = 0.050). Geographic location/district was found to be significantly associated with ART initiation (*p* < 0.001), with MSM from GP Ekurhuleni Metropolitan Municipality, MP Ehlanzeni District Municipality, KZN eThekwini Metropolitan Municipality, and KZN uMgungundlovu District Municipality showing higher odds of being initiated on ART than those from the City of Tshwane. The model further showed that the MSM had 3.514 higher odds of being initiated on ART during the COVID-19 period (aOR = 3.514; 95% CI: 2.471–4.998; *p* < 0.001). Those in the 35–44 years age group also showed 1.511 higher odds of being initiated on ART compared to those in the 16–24 years age group (aOR = 1.511; 95% CI: 1.012–2.256; *p* = 0.044).

## 4. Discussion

The current study aimed to determine the association between HIV testing modalities and ART initiation among MSM in selected provinces of South Africa. The findings of this study showed that most of the MSM were in the 25–34 years age group (N = 1672; 50.2%) and located in the KwaZulu-Natal eThekwini Metropolitan Municipality (N = 985; 29.4%), followed by Gauteng‘s Ekurhuleni Metropolitan Municipality (N = 849; 25.4%). Rapid testing (N = 1721; 51.4%) and the SNS (N = 1464; 43.8%) were the most used HIV testing modalities in this study population. This could be due to rapid testing being the standard of care and widely available, and the SNS being actively promoted, which may lead to its increased uptake among MSM living with HIV. Testing using the SNS is further preferable among key populations because of the leadership of peers whom they trust and can relate to, the provided counselling, and its association with reduced stigma [25]. It further exhibits positive role modelling. Moreover, the SNS is often incorporated into existing social structures rather than traditional health facilities, and there is sustained peer support and navigation that could be associated with greater retention in care rates, among other things [25].

The lower uptake of both HIV self-screening and index testing may be limited by the availability of test kits and logistical factors, as well as the rate of partner disclosures and the availability of index cases, respectively. Moreover, both the Chi-square test and logistic regression showed a statistically significant association between testing modalities and ART initiation. Participants who tested for HIV through the SNS (98.6%) demonstrated the highest proportions of ART initiation, as shown by the Chi-square test. Logistic regression further demonstrated that MSM who tested for HIV using the SNS had over 12 times higher odds of initiating ART compared to those who used a rapid test.

Aligning with the prevailing literature regarding the utility of community- or peer-driven mechanisms to broaden HIV diagnosis and facilitate care in key populations, Rucinski et al. [3] found that the majority of individuals among MSM and transgender women (TGW) who were tested for HIV used community-based and clinic-based strategies. Furthermore, they found that peer outreach and peer-driven index testing strategies had the highest yield in diagnoses; however, these strategies were employed less frequently than other strategies. These findings demonstrate the effectiveness of social network approaches and direct strategies to locate MSM at risk of being undiagnosed and not linked to care. Similarly, both Mahachi et al. [26] and Lillie et al. [27] noted that peer-based strategies can become saturated over time. However, peer-based strategies still provide value in engaging individuals who are at the highest risk for HIV.

In line with the WHO’s recommendations [28], incorporating social network testing interventions as an enhancement to HIV programmes may have improved the acceptability, feasibility and uptake of HIV testing among key populations who may face some stigma, discrimination, legal barriers, or other factors that may limit their health facility access. The current study supports the necessity for socially acceptable implementation of social network testing according to resource allocation and the local epidemiologic context. Along with these findings, Zhang et al. [29] indicated that social network-delivered assisted HIV self-testing achieved greater linkage to ART initiation and confirmatory testing than unassisted or clinic-based delivery. In addition, Hu et al. [30] corroborated the facilitation of HIV testing by social network strategies among key populations due to peer communication, as well as reduced social barriers to testing. Consequently, benefitting from a peer HIV service delivery system may lead to increased HIV care. Similarly to our findings, Lavoie et al. [31] reported a substantially greater proportion of ART initiation among participants diagnosed through index testing in comparison to those diagnosed through mobile or outreach testing. In addition, Rentsch et al. [32] demonstrated that same-day HIV diagnosis and linkage to care proved to be non-significant predictors of ART initiation, but the type of testing was important and significantly predicted ART initiation overall. This may suggest that there is a potential benefit to being part of a system or programme that is related to engagement in care. Furthermore, there were 3.514 higher odds of MSM being initiated on ART in the current study during the COVID-19 period. This may be due to the continuation of the MSM programme during the pandemic, along with the implementation of differentiated service delivery models such as HIV-related services being delivered to participants’ homes, with the community at the centre [23]. This is supported by Sebati et al. [23], who reported improved HIV treatment outcomes including increased HIV testing, ART initiations, and ART collections among the same MSM during the COVID-19 pandemic as compared to before.

In the current study, age group was found to be significantly associated with ART initiation, with those in the 35–44 years age group having 1.511 times higher odds of being initiated on ART than those in the 16–24 years age group. This may be due to numerous factors, including the difficulty of accepting and concerns with disclosing the diagnosis, the dual stigma of being an MSM that is living with HIV, or the financial constraints and dependency on other people. In addition to the typical barriers that MSM face, younger MSM may further lack the maturity to prioritise ART initiation due to competing interests such as education or work and a lack of support, among other factors [33,34]. Contradicting results were reported by Afrashteh [35], who reported older age to be a risk factor for late ART initiation. On the other hand, Lavoie et al. [31] found that ART initiation was associated with the mode of ART testing and the locations of residence and care. Badru et al. [36] reported that people living in urban areas had higher odds of being initiated on ART earlier because they had greater access to more health infrastructure and coordinated networks of HIV care. Also, national non-governmental organisations providing community-based HIV services in urban and peri-urban areas, such as the MSM programme from this study, may lead to increased ART initiation based on improved peer networks and accessibility. The design of the programme included peer support, home-based testing, and linkage to care, which may be closely aligned with the WHO’s Universal Test-and-Treat approach (UTT). The UTT recommends starting ART rapidly following diagnosis. As recommended by the WHO [37], early ART initiation for newly diagnosed individuals is safe, acceptable and effective in promoting reductions in onward transmission and better long-term health outcomes.

### Strengths and Limitations of the Study

A major strength of this study is its use of a large, routine programme dataset comprising over 3300 MSM across multiple high-HIV-burden provinces in South Africa, enhancing the representativeness and external validity of the findings. Furthermore, the inclusion of only MSM living with HIV/eligible for ART initiation strengthens the internal validity of the study. The data was collected using standardised Department of Health systems (REDCap and Tier.net), ensuring consistency, completeness, and alignment with national reporting indicators. The retrospective cohort design further enabled assessment of temporal relationships between HIV testing modalities and ART initiation while adjusting for key confounders such as age group, geographic location, and the COVID-19 period.

Nevertheless, the study does have some limitations. Some variables, including educational level, socioeconomic status, and behavioural factors, among others that could potentially be associated with ART initiation, were unavailable in the routine programme data analysed in the current study. Hence, the possibility of residual confounding cannot be excluded from the study. Some of the interactions observed in the analysis could be attributed to the unmeasured factors as they are all eligible confounders for both the selection of HIV testing modalities and ART initiation. Individuals using community-based HIV services may differ from those using facility-based HIV services or even across the various types of community-based HIV testing approaches in terms of health literacy, HIV knowledge, trust in the healthcare system, social capital, etc. Moreover, those utilising community-based HIV services may receive enhanced counselling, linkage support, and follow-ups [25,38]. The nature of the programmatic data, wherein the MSM were not randomly assigned to certain HIV testing modalities but rather selected which testing modality they preferred, could have introduced causal inference. Potential interaction effects could not be explored due to sample size limitations, especially for index testing and HIV self-screening, which prevented adequately powered interaction analysis in this study. However, these will be considered in future research. The participants were MSM who are part of a specific HIV prevention programme, which limits generalising the findings beyond the programme or the MSM population. Lastly, this study reported associations between ART initiation and HIV testing modalities but cannot report on causality due to its observational approach. Nevertheless, the findings contribute towards understanding the association between HIV testing modalities and ART initiation among MSM. Furthermore, this study underscores the potential of community-based and peer-driven HIV services in improving the engagement of particularly key and hard-to-reach populations in the HIV treatment cascade.

## 5. Conclusions

This study aimed to determine the association between HIV testing modalities and ART initiation among MSM in selected provinces of South Africa. The current study observed a significant association between ART initiation and HIV testing modalities. Specifically, the SNS and index testing were associated with over 12- and 3.7-fold higher odds of ART initiation, respectively, among MSM. These results further support suggestions that community-based and peer-driven HIV strategies may be associated with improved case finding, HIV testing, linkage to care, and engagement in the HIV care continuum as partially observed with the SNS in the current study. Hence, these may be scaled up alongside the conventional facility-based services. Community-based and peer-led HIV testing may further be comprehensively integrated with linkage support to enhance their reach to high-risk populations such as MSM. The variability of the results across the different districts highlights the need for context-specific and targeted interventions that take local factors such as community readiness and resource or infrastructure availability into consideration. The above may be necessary to be considered in HIV programme designs and implementation. Future research should assess this association across different key populations across the various HIV care continuum stages to develop more inclusive, age group-relevant, and context-specific HIV care delivery models.

## Figures and Tables

**Table 1:** Characteristics of men who have sex with men included in this study.

Characteristics	N	%
Age group (years)		
16–24	332	9.9
25–34	1678	50.2
35–44	1008	30.1
45+	327	9.8
Location/District		
GP City of Tshwane Metropolitan Municipality	765	22.9
GP Ekurhuleni Metropolitan Municipality	849	25.4
MP Ehlanzeni District Municipality	200	6.0
KZN eThekwini Metropolitan Municipality	985	29.4
KZN uMgungundlovu District Municipality	546	16.3
HIV testing modality		
Rapid test	1721	51.4
HIV self-screening	80	2.4
SNS	1464	43.8
Index testing	80	2.4
ART initiation within 30 days of HIV diagnosis		
No	373	11.2
Yes	2972	88.8

MP: Mpumalanga Province; GP: Gauteng Province; KZN: KwaZulu-Natal Province; ART: antiretroviral therapy; SNS: social network strategy.

**Table 2:** Chi-square tests showing the association between HIV testing modalities and antiretroviral therapy initiation among men who have sex with men.

			Testing Modality				Total	df	*p*-Value
			Rapid Test	HIV Self-Screening	SNS	Index Testing			
Initiated on ART	No	N	339	11	20	3	373	3	*p* < 0.001 ***
		%	19.7%	13.8%	1.4%	3.8%	11.2%		
	Yes	N	1382	69	1444	77	2972		
		%	80.3%	86.3%	98.6%	96.3%	88.8%		
Total			1721	80	1464	80	3345		

ART: antiretroviral therapy; degrees of freedom; SNS: social network strategy; *** *p* < 0.001.

**Table 3:** Logistic regression showing the association between HIV testing modalities and antiretroviral therapy initiation among men who have sex with men, adjusted for age group, location, and the COVID-19 period.

	df	*p*-Value	aOR	95% CI	
				Lower	Upper
HIV testing modality					
Rapid test (REF)	3	<0.001 ***	-	-	-
HIV self-screening	1	0.050	0.472	0.223	1.000
SNS	1	<0.001 ***	12.166	7.617	19.430
Index testing	1	0.029 *	3.719	1.142	12.109
Age group (in years)					
16–24 (REF)	3	0.030 *	-	-	-
25–34	1	0.066	1.426	0.977	2.081
35–44	1	0.044 *	1.511	1.012	2.256
45+	1	0.718	0.916	0.567	1.478
Location/District					
GP City of Tshwane Metropolitan Municipality (REF)	4	<0.001 ***	-	-	-
GP Ekurhuleni Metropolitan Municipality	1	<0.001 ***	5.583	3.766	8.276
MP Ehlanzeni District Municipality	1	<0.001 ***	15.231	6.094	38.066
KZN eThekwini Metropolitan Municipality	1	<0.001 ***	3.367	2.489	4.555
KZN uMgungundlovu District Municipality	1	<0.001 ***	8.077	4.896	13.325
COVID-19 period					
No (REF)	1	<0.001 ***	-	-	-
Yes	1	<0.001 ***	3.514	2.471	4.998

aOR: adjusted odds ratio; CI: confidence interval; df: degrees of freedom; REF: reference; SNS: social network strategy; *** *p* < 0.001; * *p* < 0.05.

## Data Availability

Data is available upon request from the corresponding author.

## Abbreviations

The following abbreviations are used in this manuscript:

PLHIV	People living with HIV
MSM	Men who have sex with men
SNS	Social network strategy
ART	Antiretroviral therapy
HTC	HIV testing and counselling
UTT	Universal Test-and-Treat approach
TGW	Transgender women
KZN	KwaZulu-Natal Province
MP	Mpumalanga Province
GP	Gauteng Province
